# Third-line treatment options in metastatic pancreatic cancer patients: a real-world study

**DOI:** 10.3389/fonc.2023.1251258

**Published:** 2023-09-21

**Authors:** Hong-Rui Lu, Peng-Fei Zhu, Ya-Ya Deng, Zhe-Ling Chen, Liu Yang

**Affiliations:** ^1^ Graduate School of Clinical Medicine, Bengbu Medical College, Bengbu, Anhui, China; ^2^ Cancer Center, Department of Medical Oncology, Zhejiang Provincial People's Hospital, Affiliated People's Hospital, Hangzhou Medical College, Hangzhou, Zhejiang, China; ^3^ The Qingdao University Medical College, Qingdao, Shandong, China

**Keywords:** chemotherapy, immunotherapy, pancreatic cancer, targeted therapy, third-line treatment

## Abstract

**Background:**

There are currently no standard therapy regimens for the third-line treatment of metastatic pancreatic cancer (mPC) patients. The aim of the present study was to compare the efficacy and safety of different third-line therapy regimens for mPC in the real-world.

**Methods:**

This study retrospectively analyzed mPC patients admitted to Zhejiang Provincial People’s Hospital between June 2013 and January 2023. All patients’ diagnoses were pathologically confirmed and their treatment was continued after the second-line therapy failed. The primary study endpoints included median overall survival (mOS), median progression-free survival (mPFS), and disease control rate (DCR).

**Results:**

A total of 72 patients were enrolled in the study. Of these, 36 patients received chemotherapy alone, 16 received chemotherapy combined with targeted therapy or immunotherapy, 14 received chemotherapy-free antitumor therapy, and six received palliative care. The mPFS value for these groups was 4.40 months, 5.20 months, 2.33 months, and 0.80 months, respectively. The mOS value was 6.90 months, 5.90 months, 3.33 months, and 0.80 months, respectively. The DCR was 33.4%, 31.3%, 21.4%, and 0.0%, respectively. Overall, there were significant differences in prognosis between the palliative care group and the other treatment groups (mOS, *P* < 0.001; mPFS *P* < 0.001; DCR, *P* < 0.001). The differences among the mPFS, mOS, and DCR for different antitumor therapy regimens were not statistically significant. Compared to the chemotherapy alone group, the chemotherapy combined with targeted therapy or immunotherapy group experienced more adverse events (100% vs. 75.0%; *P* = 0.002). Chemotherapy combined with targeted therapy or immunotherapy was associated with a higher risk of grade 3/4 hyperaminotransferemia compared to chemotherapy alone (31.3% vs. 0.0%; *P* = 0.020) and chemotherapy-free antitumor therapy (31.3% vs. 0.0%; *P* = 0.020).

**Conclusions:**

Third-line antitumor therapy can prolong the survival time of patients with mPC. Targeted therapy or immunotherapy failed to further improve survival benefits based on chemotherapy results. Patients who underwent the third-line treatment with good physical status and family history of cancer were independent prognostic factors for longer mOS. The sequencing of fluorouracil and gemcitabine in the front-line therapy did not affect third-line mOS.

## 1 Introduction

Pancreatic cancer (PC) is a highly aggressive tumor. Its five-year survival rate is 5%–10%, and life expectancy at diagnosis is less than 5 months (1). PC is the fourth leading cause of cancer death in the United States and the sixth in China (2, 3). Since PC occurs deep in the abdomen behind the stomach and in front of the spine, it does not cause obvious symptoms in its early stages. About 50% of patients develop metastases at initial diagnosis, which is a major factor in poor outcomes (4). Among all patients receiving first-line chemotherapy for PC, 57% went on to receive second-line therapy and 22% received third-line therapy (5).

Systemic therapy for locally advanced or metastatic disease has been documented in the National Comprehensive Cancer Network guidelines (6). FOLFIRINOX (category 1) and AG (category 1) are listed as the preferred recommended first-line chemotherapy treatments for patients in good physical condition, while gemcitabine monotherapy is recommended for patients in poor physical condition with metastatic PC (mPC) (7, 8). Almost all PC patients progress within a few months during or after first-line chemotherapy (9). Fluoropyrimidine-based regimen is the recommended subsequent treatment option for patients with a good performance status and those previously treated with gemcitabine-based therapy. Gemcitabine-based regimen is advised for patients with a good performance status and those previously treated with fluoropyrimidine-based therapy. Gemcitabine (category 1), capecitabine, and 5-fluoropyrimidine are suggested for patients with a poor performance status (10, 11). Pembrolizumab is used in an advanced disease setting as the first-line and subsequent treatment for PC patients with high microsatellite instability and mismatch repair-deficiency (12). Larotrectinib or entrectinib can be considered for *NTRK* gene fusion-positive diseases (13, 14).

Unfortunately, most patients face the challenges of tumor progression, chemotherapy resistance, and toxic effects after receiving second-line chemotherapy. Chemotherapy remains the standard of care for advanced disease. Research into novel therapies is ongoing and includes immunotherapy, targeted therapy, vaccines, and oncolytic viruses. Although most PC patients have gene mutations, there are few approved targeted therapies. New antitumor drugs for various targets are currently being developed and tested (15). PC is considered to be a ‘cold tumor’ in immunotherapy due to its typical bone marrow cell infiltration, lack of CD8+ T cells, and low activation markers. Except for 1% of patients with high microsatellite instability, PC is almost completely unsuitable for immunotherapy (16). According to the national guidelines for diagnosis and treatment of PC in China in 2022, continuing chemotherapy for PC patients who failed to respond to second-line chemotherapy is controversial, and there are no clear chemotherapy regimens to recommend (17). However, chemotherapy is still the most common choice for the third-line treatment in PC patients. There are only a handful of third-line chemotherapy drugs available, and many doctors choose to implement chemotherapy re-challenge programs for these patients (18). The efficacy and safety of various third-line treatments in PC patients are still awaiting confirmation, and clinical predictors for third-line treatment option selection are still lacking.

A considerable number of patients still have sufficient physical strength to receive antitumor therapy when the disease progresses to the third-line stage. The present study aimed to compare the efficacy and safety of different third-line therapies for mPC. The efficacy, safety, and relevance of various combinations of third-line antitumor therapies, including chemotherapy, targeted therapy, and immunotherapy, were explored in order to investigate the status of third-line therapy in mPC.

## 2 Patients and methods

### 2.1 Patients

We analyzed 72 patients with mPC who received third-line therapy and were admitted to Zhejiang Provincial People’s Hospital between June 2013 and January 2023. Clinical patient staging was performed according to the American Joint Committee on Cancer guidelines. All procedures were carried out in accordance with the ethical standards of the Committee on Human Experimentation (institutional and national) and the Declaration of Helsinki. The study was approved by the Ethics Committee of Zhejiang Provincial People’s Hospital.

### 2.2 Therapy schedule

The common chemotherapy regimens for third-line treatment are FOLFIRINOX (oxaliplatin 85 mg/m^2^, irinotecan 150 mg/m^2^, leucovorin 400 mg/m^2^, and 5-fluorouracil 2400 mg/m^2^ administered every two weeks), FOLFIRI (irinotecan 180 mg/m^2^, leucovorin 400 mg/m^2^, and 5-fluorouracil 2400 mg/m^2^ administered every two weeks), AG (albumin-bound paclitaxel 125 mg/m^2^ on days 1 and 8 and gemcitabine 1,000 mg/m^2^ on days 1 and 8 administered every three weeks), GS (gemcitabine 1,000 mg/m^2^ on days 1 and 8 and S-1 60 mg twice daily on days 1–14 administered every three weeks), CapeOX (oxaliplatin 135 mg/m^2^ and capecitabine 1000 mg twice daily on days 1–14 administered every three weeks), GX (gemcitabine 1,000 mg/m^2^ on days 1 and 8 and capecitabine 830 mg twice daily on days 1–14 administered every three weeks), AS (albumin-bound paclitaxel 125 mg/m^2^ on days 1 and 8 and S-1 60 mg twice daily on days 1–14 administered every three weeks), gemcitabine (gemcitabine 1,000 mg/m^2^ on days 1 and 8 administered every three weeks), and S-1 (S-1 60 mg twice daily on days 1–14 administered every three weeks). Pembrolizumab (200 mg administered every three weeks) is a common programmed cell death protein 1 for third-line treatment. Apatinib (500 mg administered every day) is a common targeted drug. Clinicians adjusted the drug dose according to the patient’s adverse events (AEs) experienced during therapy.

### 2.3 Assessment

The tumor response was evaluated based on the revised Response Evaluation Criteria in Solid Tumors (version 1.1) using computed tomography and magnetic resonance imaging every 2–3 treatment cycles. The AEs were evaluated according to the Common Terminology Criteria for Adverse Events (version 5.0).

### 2.4 Statistical analyses

The median overall survival (mOS) and median progression-free survival (mPFS) rates were the primary endpoints. The disease control rate (DCR) and AEs were the secondary endpoints. All of the statistical analyses were performed using IBM SPSS Statistics software version 26.0 (IBM Corp., Grouponk, NY, NY, USA). Kaplan–Meier method was used to analyze the OS and PFS. Cox proportional regression model was used to analyze the survival and prognosis. Significant factors (*P* < 0.1) identified using univariate Cox regression analysis were included in multivariate Cox regression analysis. T-test was used for AE comparison between groups.

## 3 Results

### 3.1 Efficacy and survival analysis of third-line treatment

#### 3.1.1 Clinical factors for patients

Baseline characteristics of mPC patients who received the third-line treatment are shown in **Table 1**. A total of 72 patients were enrolled in the study, of which 36 received chemotherapy alone, 16 received chemotherapy combined with targeted therapy or immunotherapy, 14 received chemotherapy-free antitumor therapy, and six received palliative treatment. Patient characteristics were not balanced between each group, including the baseline of Eastern Cooperative Oncology Group Performance status (ECOG PS), first-line treatment, and second-line treatment.

**Table 1:** Patient baseline characteristics of third-line treatment.

Variables	Chemotherapy alone		Chemotherapy combined with targeted or immunotherapy		Chemotherapy-free antitumor therapy		Alleviative treatment		total		p value
	(n=36)		(n=16)		(n=14)		(n=6)		(n=72)		
	No.	%	No.	%	No.	%	No.	%	No.	%	
**Sex**											0.247
Male	24	66.7	7	43.8	11	78.6	4	66.7	46	63.9	
Female	12	33.3	9	56.2	3	21.4	2	33.3	26	36.1	
**Median age (range)**	61.75±7.632		62.38±9.959		64.00±10.627		60.00±13.023		62.18±9.135		0.861
**BMI**											0.830
thin	4	11.1	3	18.8	2	14.3	2	33.3	11	15.3	
healthy	30	83.3	12	75.0	11	78.6	4	66.7	57	79.2	
overweight	1	2.8	1	6.2	1	7.1	0	0.0	3	4.1	
obesity	1	2.8	0	0.0	0	0.0	0	0.0	1	1.4	
**ECOG PS**											0.030
0-1	26	72.2	8	50.0	5	35.7	0	0.0	39	54.2	
2-5	10	27.8	8	50.0	9	64.3	6	100.0	33	45.8	
**First-line treatment**											0.030
G-based	24	66.7	10	62.5	3	21.4	4	66.7	41	56.9	
F-based	12	33.3	6	37.5	11	78.6	2	33.3	21	43.1	
**First-line PFS (months) (range)**	5.75 (2.55-8.32)		3.28 (1.98-7.08)		6.55 (3.25-14.08)		2.90 (1.77-7.87)		5.37 (2.10-8.21)		0.241
**Second-line treatment**											0.027
F-based	25	69.4	8	50.0	5	35.7	6	100.0	44	61.1	
G-based	11	30.6	6	37.5	8	57.1	0	0.0	25	34.7	
Other	0	0.0	2	12.5	1	7.2	0	0.0	3	4.2	
**Second-line PFS (months) (range)**	5.03 (2.24-7.83)		4.52 (2.75-7.22)		5.10 (1.63-9.56)		2.52 (0.79-3.99)		4.38 (2.24-7.83)		0.218
**First and second line treatment order**											0.215
G-based to F-based	17	47.2	7	43.7	3	21.4	4	66.7	31	43.1	
G-based to G-based	6	16.7	1	6.3	1	7.2	0	0.0	8	11.1	
G-based to Other	1	2.7	2	12.4	0	0.0	0	0.0	3	4.2	
F-based to G-based	6	16.7	5	31.3	6	42.9	0	0.0	17	23.6	
F-based to F-based	6	16.7	1	6.3	3	21.4	2	33.3	12	16.7	
F-based to Other	0	0.0	0	0.0	1	7.1	0	0.0	1	1.3	
**Sum of first and second line PFS (range)**	11.77 (7.17-15.00)		10.70 (6.00-12.28)		14.67 (6.91-27.11)		5.10 (2.95-12.11)		11.00 (6.63-14.80)		0.085
**Family history of cancer**											0.592
Yes	7	19.4	2	12.5	4	28.6	2	33.3	14	19.4	
No	29	80.6	14	87.5	10	71.4	4	66.7	58	80.6	
**Tumor location**											0.219
Head	12	33.3	10	62.5	5	35.7	5	83.3	32	44.4	
Body	0	0.0	0	0.0	1	7.1	0	0.0	1	1.4	
Tail	3	8.3	2	12.5	1	7.1	0	0.0	6	8.4	
Head+body	1	2.8	0	0.0	0	0.0	0	0.0	1	1.4	
Body+tail	20	55.6	4	25.0	7	50.1	1	16.7	32	44.4	
**Metastatic type**											
Liver	24	66.7	12	75.0	11	78.6	2	33.3	49	68.1	0.238
Peritoneal	22	61.1	8	50.0	7	50.0	2	33.3	39	54.2	0.595
Lung	5	13.9	1	6.3	1	7.1	1	16.7	8	11.1	0.751
Distant lymph node	3	8.3	2	12.5	4	28.6	0	0.0	9	12.5	0.273
**Previous surgery**											0.158
Yes	20	55.6	4	25.0	6	42.9	4	66.7	34	47.2	
No	16	44.4	12	75.0	8	57.1	2	33.3	38	52.8	
**CEA**											0.360
Normal	16	44.4	3	18.8	5	35.7	2	33.3	26	36.1	
Abnormal	20	55.6	13	81.2	9	64.3	4	66.7	46	63.9	
**CA125**											0.090
Normal	21	58.3	6	37.5	3	21.4	2	33.3	32	44.4	
Abnormal	15	41.7	10	62.5	11	78.6	4	66.7	40	55.6	
**CA199**											>0.999
Normal	7	19.4	3	18.8	3	21.4	1	16.7	14	19.4	
Abnormal	29	80.6	13	81.2	11	78.6	5	83.3	58	80.6	
**NLR**											0.983
Normal	20	55.6	9	56.3	8	57.1	4	66.7	41	56.9	
Abnormal	16	44.4	7	43.7	6	42.9	2	33.3	31	43.1	
**PLR**											0.801
Normal	22	61.1	11	68.8	9	64.3	5	83.3	47	65.3	
Abnormal	14	38.9	5	31.2	5	35.7	1	16.7	25	34.7	

BMI, Body Mass Index; ECOG PS, Eastern Cooperative Oncology Group Performance status; G-based, Gemcitabine based therapy; F-based, Fluorouracil based treatment; PFS, Progression-free survival; NLR, Neutrophil to lymphocyte ratio; PLR, Platelet to lymphocyte ratio; MLR, Monocyte to lymphocyte ratio.

#### 3.1.2 Efficacy

**Figure 1:**
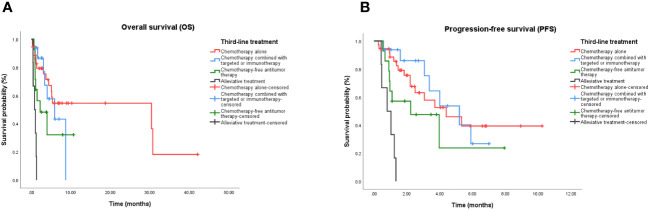
Kaplan-Meier curve in patients treated with third-line treatment. **(A)** OS in patients treated with chemotherapy alone, chemotherapy combined with targeted or immunotherapy, chemotherapy-free antitumor therapy, and palliative care. **(B)** PFS in patients treated with chemotherapy alone, chemotherapy combined with targeted or immunotherapy, chemotherapy-free antitumor therapy, and palliative care. OS, Overall survival; PFS, Progression-free survival.

The mOS values for the chemotherapy alone, chemotherapy combined with targeted therapy or immunotherapy, chemotherapy-free antitumor therapy, and palliative treatment groups were 6.9 months (95% confidence interval (CI), 0.5–13.9 months), 5.9 months (95% CI, 1.6–10.2 months), 3.3 months (95% CI, 0.2–5.0 months), and 0.8 months (95% CI, 0.1–1.5 months), respectively. The mPFS values were 4.4 months (95% CI, 1.8–7.0 months), 5.2 months (95% CI, 2.7–7.7 months), 2.3 months (95% CI, 0.3–4.6 months), and 0.8 months (95% CI, 0.1–1.5 months), respectively. Kaplan–Meier analysis showed that the mOS and mPFS values in mPC patients who received chemotherapy alone (*P <* 0.001; *P <* 0.001), chemotherapy combined with targeted therapy or immunotherapy (*P <* 0.001; *P <* 0.001), and chemotherapy-free antitumor therapy (*P <* 0.001; *P <* 0.001) were greater than those in patients who received palliative treatment. There was no statistical difference in mOS and mPFS between groups of mPC patients who received antitumor therapy, chemotherapy alone, and chemotherapy combined with targeted therapy or immunotherapy (*P* = 0.588; *P* = 0.783), chemotherapy alone and chemotherapy-free antitumor therapy (*P* = 0.061; *P* = 0.189), chemotherapy combined with targeted therapy or immunotherapy and chemotherapy-free antitumor therapy (*P* = 0.265; *P* = 0.154; **Figure 1**).

The DCRs for the chemotherapy alone, chemotherapy combined with targeted therapy or immunotherapy, chemotherapy-free antitumor therapy, and palliative treatment groups were 33.4%, 31.3%, 21.4%, and 0.0%, respectively. The DCRs for mPC patients who received chemotherapy alone (*P <* 0.001), chemotherapy combined with targeted therapy or immunotherapy (*P <* 0.001), and chemotherapy-free antitumor therapy (*P <* 0.001) were higher than those for patients who received palliative treatment. There was no statistical difference in DCRs between mPC patients who received antitumor therapy, chemotherapy alone, and chemotherapy combined with targeted therapy or immunotherapy (*P* = 0.565), chemotherapy alone and chemotherapy-free antitumor therapy (*P* > 0.999), chemotherapy combined with targeted therapy or immunotherapy and chemotherapy-free antitumor therapy (*P* > 0.999; **Table 2**).

**Table 2:** Rates of response in patients of third-line treatment.

Variables	Chemotherapy alone		Chemotherapy combined with targeted or immunotherapy		Chemotherapy-free antitumor therapy		Alleviative treatment		total		p value
	(n=36)		(n=16)		(n=14)		(n=6)		(n=72)		
	No.	%	No.	%	No.	%	No.	%	No.	%	
Response											
Partial response	1	2.8	1	6.3	1	7.1	0	0.0	3	4.2	
Stable disease	11	30.6	4	25.0	2	14.3	0	0.0	17	23.6	
Progressive disease	24	66.7	11	68.8	11	78.6	6	100.0	52	72.2	
**Disease control rate**	12	33.4	5	31.3	3	21.4	0	0.0	20	27.8	0.396

#### 3.1.3 Cox proportional hazards regression analysis

**Figure 2:**
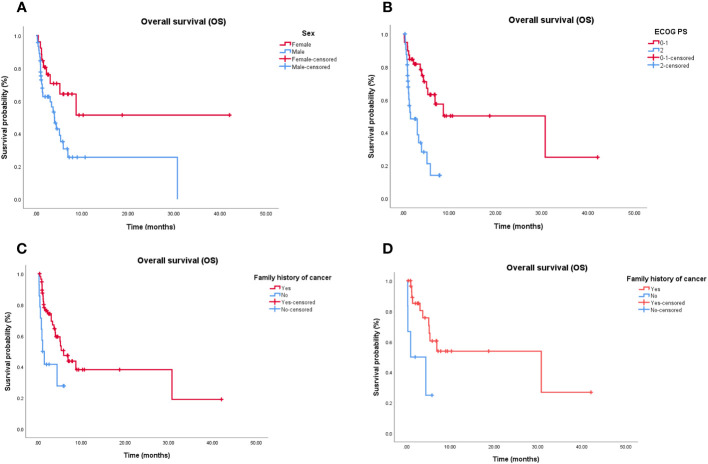
Independent significant factors of long-term survival in third-line treatment and chemotherapy alone treatment. **(A)** Women treated with third-line treatment have longer OS than man (*P* = 0.006). **(B)** Patients treated with third-line treatment, with ECOG PS 0-1 have longer OS than patients with ECOG PS ≥2 (*P* < 0.001). **(C)** Patients treated with third-line treatment, with family history of cancer have longer OS than patients without family history of cancer (*P* = 0.035). **(D)** Patients treated with chemotherapy alone, with family history of cancer have longer OS than patients without family history of cancer (*P* = 0.021). OS, Overall survival; ECOG PS, Eastern Cooperative Oncology Group Performance status.

Cox proportional hazards models and Kaplan–Meier analysis used for patients undergoing the third-line treatment showed that female patients (**Figure 2A**), patients with ECOG PS 0–1 (**Figure 2B**), and those with family history of cancer (**Figure 2C**) were more likely to respond to the third-line treatment (**Supplementary Table 1**). Patients with family history of cancer were particularly suitable for the chemotherapy alone regimen (**Supplementary Table 2**; **Figure 2D**). There was no independent factor in multivariate Cox proportional hazards models of patients treated with chemotherapy combined with targeted therapy or immunotherapy (**Supplementary Table 3**) or with chemotherapy-free antitumor therapy (**Supplementary Table 4**).

#### 3.1.4 Safety

The AE data for 72 patients are listed in **Table 3**. Most patients (63, 87.5%) experienced different degrees of AEs, and more than half of patients experienced grade 3/4 AEs (39, 54.2%). Compared to the chemotherapy alone group, chemotherapy combined with targeted therapy or immunotherapy group experienced more AEs (100.0% vs. 75.0%; *P* = 0.002). However, there was no statistical difference between the two groups in grade ¾ AEs (75.0% vs. 47.2%; *P* = 0.056). Compared to the chemotherapy alone (0.0% vs. 31.3%; *P* = 0.020) and chemotherapy-free antitumor therapy (0.0% vs. 31.3%; *P* = 0.020) groups, the chemotherapy combined with targeted therapy or immunotherapy group experienced more grade 3/4 leukopenia.

**Table 3:** Rates of AEs in patients of third-line treatment.

AEs	Chemotherapy alone		Chemotherapy combined with targeted or immunotherapy		Chemotherapy-free antitumor therapy		Alleviative treatment	
	Any grade (%)	Grade 3/4 (%)	Any grade (%)	Grade 3/4 (%)	Any grade (%)	Grade 3/4 (%)	Any grade (%)	Grade 3/4 (%)
Leukopenia	9 (25.0)	0	12 (75.0)	5 (31.3)	2 (14.3)	0	1 (16.7)	0
Thrombocytopenia	4 (11.2)	2 (5.6)	5 (31.2)	3 (18.8)	3 (21.4)	2 (14.3)	0	0
Anemia	13 (36.2)	2 (5.6)	8 (50.0)	2 (12.5)	3 (21.4)	0	2 (33.3)	2 (33.3)
Neutropenia	3 (8.4)	2 (5.6)	3 (18.8)	0	5 (35.7)	3 (21.4)	1 (16.7)	0
Vomiting	7 (19.4)	2 (5.6)	4 (25.0)	2 (12.5)	4 (28.6)	1 (7.1)	1 (16.7)	1 (16.7)
Diarrhea	5 (13.9)	3 (8.3)	2 (12.5)	0	3 (21.4)	2 (14.3)	0	0
Hyperbilirubinemia	10 (27.8)	4 (11.1)	7 (43.7)	3 (18.8)	3 (21.4)	1 (7.1)	0	0
Hyperaminotransferemia	2 (5.6)	0	3 (18.8)	1 (6.3)	1 (7.1)	0	1 (1.4)	0
Hyperalkaline phosphatinemia	10 (27.8)	4 (11.1)	8 (50.0)	4 (25.0)	3 (21.4)	2 (14.3)	3 (50.0)	1 (16.7)
Hypercreatinemia	5 (13.9)	0	4 (25.0)	0	1 (7.1)	0	2 (33.3)	1 (16.7)
Proteinuria	4 (11.1)	0	6 (37.5)	0	3 (11.4)	1 (7.1)	0	0
Hematuria	2 (5.6)	0	3 (18.7)	2 (12.5)	1 (7.1)	0	0	0
Total	27 (75.0)	17 (47.2)	16 (100.0)	12 (75.0)	11 (78.6)	6 (42.9)	5 (83.3)	4 (66.6)

AEs, Adverse Events.

#### 3.1.5 Dosage and survival

In our study, patients were treated with a complex chemotherapy regimen. Due to adverse reactions and physical conditions, some patients could not undergo adequate chemotherapy during the third-line treatment. Therefore, in order to further analyze the relationship between dosage and survival, we selected the most common chemotherapy regimen, the AG regimen (including combination targeting or immunotherapy regimens), as the study subjects. Among the 72 patients, 12 patients received the AG regimen as third-line treatment. Among them, 5 patients received full-dose chemotherapy, while 7 patients received reduced-dose chemotherapy. The dosage cannot be considered an independent prognostic factor for the survival of AG-treated patients (HR, 0.173; 95% CI,0.016 – 1.903; P = 0.151).

### 3.2 Subgroup analysis of efficacy and survival analysis in patients who received chemotherapy-based treatment

In the present study, most patients (52, 72.2%) received chemotherapy-based regimens as the third-line treatment. There was no difference in survival time between the chemotherapy alone and chemotherapy combined with targeted therapy or immunotherapy groups. However, the latter had a higher adverse reaction risk. Based on this, the study patients were further stratified according to the chemotherapy regimen to determine the most appropriate treatment intensity for patients receiving third-line therapy.

#### 3.2.1 Clinical factors of patients receiving chemotherapy-based treatment

Baseline characteristics of mPC patients receiving third-line chemotherapy-based treatment are shown in **Table 4**. Of the 52 patients, 12 received single-agent chemotherapy, 24 received multi-agent chemotherapy, six received single-agent chemotherapy combined with targeted therapy or immunotherapy, and 10 received multi-agent chemotherapy combined with targeted therapy or immunotherapy. The baseline characteristics, including ECOG PS, tumor site, tumor markers, and other factors, were balanced.

**Table 4:** Patient baseline characteristics of chemotherapy-based treatment.

Variables	Single-agent chemotherapy		Multi-agent chemotherapy		Single-agent chemotherapy combined with targeted/immunotherapy		Multi-agent chemotherapy combined with targeted/immunotherapy		total		p value
	(n=12)		(n=24)		(n=6)		(n=10)		(n=52)		
	No.	%	No.	%	No.	%	No.	%	No.	%	
**Sex**											0.359
Male	7	58.3	17	70.8	3	50.0	4	40.0	31	59.6	
Female	5	41.7	7	29.2	3	50.0	6	60.0	21	40.4	
**Median age** (range)	63.00±7.224		61.13±7.903		63.33±5.007		61.80±12.264		61.94±8.321		0.930
**BMI**											0.490
thin	0	0.0	4	16.7	2	33.3	1	10.0	7	13.5	
healthy	12	100.0	18	75.0	4	66.7	8	80.0	42	80.8	
overweight	0	0.0	1	4.2	0	0.0	1	10.0	2	3.8	
obesity	0	0.0	1	4.2	0	0.0	0	0.0	1	1.9	
**ECOG PS**											0.111
0-1	6	50.0	20	83.3	1	16.7	7	70.0	29	55.8	
2-5	6	50.0	4	16.7	5	83.3	3	30.0	23	44.2	
**First-line treatment**											0.532
G-based	9	75.0	15	62.5	5	83.3	5	50.0	34	65.4	
F-based	3	25.0	9	37.5	1	16.7	5	50.0	18	34.6	
**First-line PFS (months) (range)**	6.30 (4.93-7.71)		5.45 (2.10-8.23)		2.42 (1.56-8.03)		2.22 (1.73-9.24)		5.52 (2.05-7.70)		0.256
**Second-line treatment**											0.437
F-based	8	66.7	17	70.8	4	66.7	4	40.0	32	61.5	
G-based	4	33.3	7	29.2	1	16.7	5	50.0	17	32.7	
Other	0	0.0	0	0.0	1	16.6	1	10.0	3	5.8	
**Second-line PFS (months) (range)**	5.97 (2.21-8.21)		3.82 (2.19-5.83)		5.82 (3.08-12.63)		8.23 (1.98-11.41)		4.82 (2.22-8.08)		0.340
**First and second line treatment order**											0.358
G-based to F-based	6	50	11	45.8	1	16.7	6	60.0	24	46.2	
G-based to G-based	3	25	3	12.5	0	0.0	1	10.0	7	13.5	
G-based to Other	0	0.0	1	4.2	2	33.3	0	0.0	3	5.8	
F-based to G-based	2	16.7	4	16.7	3	50.0	2	20.0	11	21.2	
F-based to F-based	1	8.3	5	20.8	0	0.0	1	10.0	7	13.5	
**Sum of first and second line PFS (range)**	11.98 (7.16-17.27)		9.37 (5.28-14.14)		7.55 (5.65-20.83)		13.20 (3.91-18.53)		10.62 (6.67-14.73)		0.749
**Family history of cancer**											0.182
Yes	1	8.3	6	25.0	2	33.3	0	0.0	8	15.7	
No	11	91.7	18	75.0	4	66.7	10	100.0	43	84.3	
**Tumor location**											0.229
Head	5	41.7	7	29.1	4	66.7	6	60.0	22	42.3	
Tail	2	16.6	1	4.2	0	0.0	2	20.0	5	9.6	
Head+body	0	0.0	1	4.2	0	0.0	0	0.0	1	1.9	
Body+tail	5	41.7	15	62.5	2	33.3	2	20.0	24	46.2	
**Metastatic type**											
Liver	8	66.7	16	66.7	3	50.0	9	90.0	36	69.2	0.347
Peritoneal	7	58.3	15	62.5	4	66.7	4	40.0	30	57.7	0.711
Lung	2	16.7	3	12.5	0	0.0	1	10.0	6	11.5	0.930
Distant lymph node	0	0.0	3	12.5	0	0.0	2	20.0	5	9.6	0.375
**Previous surgery**											0.128
Yes	5	41.7	15	62.5	2	33.3	2	20.0	24	46.2	
No	7	58.3	9	37.5	4	66.7	8	80.0	28	53.8	
**CEA**											0.073
Normal	3	25.0	13	54.2	2	33.3	1	0.0	18	34.6	
Abnormal	9	75.0	11	45.8	4	66.7	9	100.0	34	65.4	
**CA125**											0.067
Normal	5	41.7	16	66.7	4	66.7	2	20.0	27	51.9	
Abnormal	7	58.3	8	33.3	2	33.3	8	80.0	25	48.1	
**CA199**											0.455
Normal	1	8.3	6	25.0	2	33.3	1	10.0	10	19.2	
Abnormal	11	91.7	18	75.0	4	66.7	9	90.0	42	80.8	
**NLR**											0.420
Normal	9	75.00	11	45.8	3	50.00	6	60.0	29	55.8	
Abnormal	3	25.00	13	54.2	3	50.00	4	40.0	23	44.2	
**PLR**											0.644
Normal	9	75	13.00	54.2	4	66.7	7	70.0	33	63.5	
Abnormal	3	25	11.00	45.8	2	33.3	3	30.0	19	36.5	

BMI, Body Mass Index; ECOG PS, Eastern Cooperative Oncology Group Performance status; G-based, Gemcitabine based therapy; F-based, Fluorouracil based treatment; PFS, Progression-free survival; NLR, Neutrophil to lymphocyte ratio; PLR, Platelet to lymphocyte ratio; MLR, Monocyte to lymphocyte ratio.

#### 3.2.2 Efficacy

**Figure 3:**
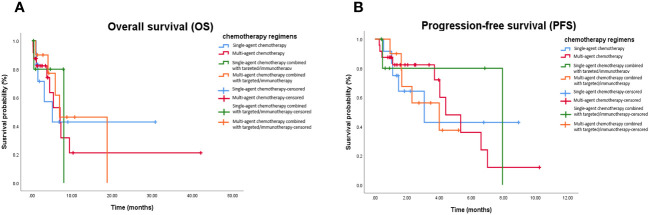
Kaplan-Meier curve in patients received chemotherapy-based treatment. **(A)** OS in patients treated with single-agent chemotherapy, multi-agent chemotherapy, single-agent chemotherapy combined with targeted or immunotherapy, and multi-agent chemotherapy combined with targeted or immunotherapy. **(B)** PFS in in patients treated with single-agent chemotherapy, multi-agent chemotherapy, single-agent chemotherapy combined with targeted or immunotherapy, and multi-agent chemotherapy combined with targeted or immunotherapy. OS, Overall survival; PFS, Progression-free survival.

The mOS values for the single-agent chemotherapy, multi-agent chemotherapy, single-agent chemotherapy combined with targeted therapy or immunotherapy, and multi-agent chemotherapy combined with targeted therapy or immunotherapy groups were 5.1 months (95% CI, 1.3–10.7 months), 7.9 months (95% CI, 1.3–8.9 months), 7.0 months (95% CI, 0.7–7.5 months), and 6.9 months (95% CI, 2.6–10.2 months), respectively. The mPFS values were 3.1 months (95% CI, 1.0–7.3 months), 6.9 months (95% CI, 1.8–9.2 months), 4.4 months (95% CI, 1.9–6.9 months), and 4.0 months (95% CI, 1.7–5.1 months), respectively. Kaplan–Meier analysis showed that there was no statistical difference in mOS and mPFS between mPC patients who received chemotherapy-based regimens, single-agent chemotherapy, and multi-agent chemotherapy (*P* = 0.967; *P* = 0.991), single-agent chemotherapy and single-agent chemotherapy combined with targeted therapy or immunotherapy (*P* = 0.951; *P* = 0.955), multi-agent chemotherapy and multi-agent chemotherapy combined with targeted therapy or immunotherapy (*P* = 0.809; *P* = 0.589), single-agent chemotherapy combined with targeted therapy or immunotherapy and multi-agent chemotherapy combined with targeted therapy or immunotherapy (*P* = 0.583; *P* = 0.416; **Figure 3**).

DCRs for the single-agent chemotherapy, multi-agent chemotherapy, single-agent chemotherapy combined with targeted therapy or immunotherapy, and multi-agent chemotherapy combined with targeted therapy or immunotherapy were 50.0%, 25.0%, 33.3%, and 40.0%, respectively. There was no statistical difference in DCRs between the mPC patients who received chemotherapy-based regimens, single-agent chemotherapy, and multi-agent chemotherapy (*P* = 0.182), single-agent chemotherapy and single-agent chemotherapy combined with targeted therapy or immunotherapy group (*P >* 0.999), multi-agent chemotherapy and multi-agent chemotherapy combined with targeted therapy or immunotherapy (*P* = 0.400), single-agent chemotherapy combined with targeted therapy or immunotherapy and multi-agent chemotherapy combined with targeted therapy or immunotherapy (*P* = 0.400; **Table 5**).

**Table 5:** Rates of response in patients of chemotherapy-based treatment.

Variables	Single-agent chemotherapy		Multi-agent chemotherapy		Single-agent chemotherapy combined with targeted/immunotherapy		Multi-agent chemotherapy combined with targeted/immunotherapy		total		p value
	(n=12)		(n=24)		(n=6)		(n=10)		(n=52)		
	No.	%	No.	%	No.	%	No.	%	No.	%	
Response											
Partial response	0	0.0	2	8.3	1	16.7	0	0.0	3	5.8	
Stable disease	6	50.0	4	16.7	1	16.7	4	40.0	15	28.8	
Progressive disease	6	50.0	18	75.0	4	66.6	6	60.0	34	65.4	
**Disease control rate**	9	50.0	6	25.0	2	33.3	4	40.0	34	34.6	0.250

#### 3.2.3 Cox proportional hazards regression analysis

**Figure 4:**
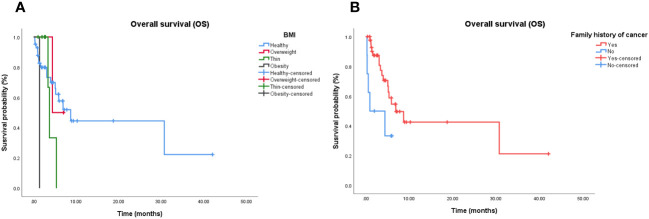
Independent significant factors of long-term survival in chemotherapy-based treatment. **(A)** Patients treated with chemotherapy-based treatment, with normal BMI have longer OS than patients with abnormal BMI (*P* = 0.021). **(B)** Patients treated with chemotherapy-based treatment, with family history of cancer have longer OS than patients without family history of cancer (*P* = 0.019). BMI, Body Mass Index; OS, Overall survival.

Cox proportional hazards models and Kaplan–Meier analysis used for patients undergoing chemotherapy-based regimens showed that patients with a normal body mass index (**Figure 4A**) and family history of cancer (**Figure 4B**) were more likely to respond to chemotherapy-based regimens (**Supplementary Table 5**). There was no independent factor in multivariate Cox proportional hazards models of patients treated with multi-agent chemotherapy (**Supplementary Table 6**). Due to the small sample size, Cox analysis was not applicable to the other three subgroups.

#### 3.2.4 Safety

AEs were assessed in 52 patients (**Table 6**). Most patients (42, 80.8%) experienced different degrees of AEs, and some patients experienced grade 3/4 AEs (20, 38.5%). Compared to the single-agent chemotherapy group, single-agent chemotherapy combined with targeted therapy or immunotherapy group experienced more AEs (100.0% vs. 66.7%; *P* = 0.039). However, there was no significant difference between the two groups in grade 3/4 AEs (50.0% vs. 16.7%; *P* = 0.153). Compared to the multi-agent chemotherapy group, multi-agent chemotherapy combined with targeted therapy or immunotherapy group experienced more AEs (100.0% vs. 75.0%; *P* = 0.011) and more grade 3/4 leukopenia (30.0% vs. 0.0%; *P* = 0.037). However, there was no significant difference between the two groups in the total incidence of grade 3/4 AEs (70.0% vs. 33.3%; *P* = 0.517).

**Table 6:** Rates of AEs in patients of chemotherapy-based treatment.

Events	Single-agent chemotherapy		Multi-agent chemotherapy		Single-agen chemotherapy combined with targeted/immunotherapy		Multi-agent chemotherapy combined with targeted/immunotherapy	
	Any grade (%)	Grade 3/4 (%)	Any grade (%)	Grade 3/4 (%)	Any grade (%)	Grade 3/4 (%)	Any grade (%)	Grade 3/4 (%)
Leukopenia	2 (16.7)	0	4 (16.7)	0	3 (50.0)	1 (16.7)	5 (50.0)	3 (30.0)
Thrombocytopenia	1 (8.3)	0	1 (4.2)	0	0	0	2 (20.0)	0
Anemia	5 (41.7)	0	8 (33.3)	2 (8.3)	3 (50.0)	1 (16.7)	5 (50.0)	1 (10.0)
Neutropenia	1 (8.3)	1 (8.3)	1 (4.2)	0	0	0	3 (30.0)	0
Vomiting	0	0	5 (20.8)	0	1 (16.7)	0	1 (10.0)	0
Diarrhea	0	0	1 (4.2)	0	1 (16.7)	0	0	0
Hyperbilirubinemia	5 (41.7)	0	5 (20.8)	4 (16.7)	3 (50.0)	1 (16.7)	4 (40.0)	2 (20.0)
Hyperaminotransferemia	2 (16.7)	0	0	0	0	0	3 (30.0)	1 (10.0)
Hyperalkaline phosphatinemia	2 (16.7)	1 (8.3)	8 (33.3)	3 (12.5)	2 (33.3)	1 (16.7)	6 (60.0)	3 (30.0)
Hypercreatinemia	2 (16.7)	0	4 (16.7)	0	1 (16.7)	0	4 (40.0)	0
Proteinuria	0	0	4 (16.7)	0	1 (16.7)	0	5 (50.0)	0
Hematuria	1 (8.3)	0	1 (4.2)	0	0	0	1 (10.0)	0
Total	8 (66.7)	2 (16.7)	18 (75.0)	8 (33.3)	6 (100.0)	3 (50.0)	10 (100.0)	7 (70.0)

AEs, Adverse Events.

## 4 Discussion

In this study, the third-line antitumor treatment was demonstrated to benefit patients and prolong their survival time compared to palliative care. Baseline characteristics were analyzed in all patients to identify efficacy predictors. Results showed that female patients, those with ECOG PS 0–1, and patients with family history of cancer were independent prognostic factors for longer OS in a group of mPC patients who received the third-line treatment. In particular, patients with a normal body mass index and family history of cancer were independent prognostic factors for longer OS in a group of mPC patients who received chemotherapy-based treatment. This indicates that not all patients are suitable for third-line antitumor therapy and some screening is still needed. Many retrospective studies have concluded that ECOG PS is an independent prognostic factor associated with treatment efficacy. Most notably, a family history of cancer was an independent factor for longer survival time among different treatment regimes in our study. Existing research studies have reported that family history of *BRCA*-related tumors may correlate with the response to chemotherapy and OS in PC, which is similar to the results of our study (19). This may be related to genetic differences and lifestyle changes. Patients with a family history of tumor disease may have some genetic mutations and are more likely to have malignant changes when affected by the external environment compared to those without a family history (20). In addition, it has been suggested that young patients who are aware of their family history may adopt healthy behaviors, such as opportunistic screening, and/or make healthy lifestyle changes, thereby improving their prognosis (21). Surprisingly, female patients were more likely to benefit from third-line treatments for mPC. Patient characteristics were not balanced between each group, including ECOG PS, first-line treatment, and second-line treatment. In order to determine the cause of this imbalance at baseline, a review of the case data revealed that patients with ECOG PS of ≥ 2 had a poor physical performance and were more inclined to choose chemotherapy-free regimens before the third-line treatment, while patients with a better physical performance were more suitable for chemotherapy. Patients receiving palliative treatment with ECOG PS of ≥ 2 at the beginning of the second-line treatment only received fluorouracil single-agent in the second-line treatment. A significant proportion of patients (24, 33.3%) received gemcitabine-based regimen as the first-line treatment and fluorouracil-based regimen as the second-line treatment.

Since our research data and previous studies have shown that third-line antitumor therapy can bring survival benefits to patients, it was necessary to determine whether chemotherapy combined with other treatments can further improve treatment efficacy. With the recent development of novel therapies, such as immunotherapy and targeted therapy, some clinicians have chosen to combine these therapies with chemotherapy or to directly use chemotherapy-free therapies when selecting third-line treatments. This is the first real-world study to compare the efficacy and safety of various third-line treatments for advanced PC. Although there was no statistical difference in P value, Kaplan-Meier analysis showed that the chemotherapy alone (mOS, 6.9 months; 95% CI, 0.5–13.9 months) and chemotherapy combined with targeted therapy or immunotherapy (mOS, 5.9 months; 95% CI, 1.6–10.2 months) groups had a better OS compared to the chemotherapy-free group (mOS, 3.3 months; 95% CI, 0.2–5.0 months). This may be due to the insufficient sample size in the study. This investigation demonstrated for the first time that the combined targeting/immunotherapy based on chemotherapy cannot improve third-line mOS (6.9 months vs. 5.9 months, *P* = 0.588) compared to chemotherapy alone. This notion has previously been introduced in other studies investigating the first-line treatment. Previous research has revealed that PC promotes an immunosuppressive microenvironment through formation of dense stromal desmoplasia, and concurrent administration of gemcitabine plus nab-paclitaxel was poised to improve immunotherapy drug access to tumor cells via structural disruption/remodeling of the PC tumor microenvironment (22). Negative results in the CCTG PA.7 trial indicated that it was not sufficient to increase immunotherapy efficacy in the overall patient population. The CCTG PA.7 trial demonstrated no survival benefits from adding durvalumab and tremelimumab to gemcitabine and nab-paclitaxel as the first-line therapy in an unselected population of patients with PC (mOS, 9.8 months vs. 8.8 months, *P* = 0.72). Moreover, the combination of epidermal growth factor receptor inhibitors and chemotherapy failed to achieve the preclinical model estimates. In preclinical models of PC, ibrutinib combined with gemcitabine increased the levels of effector CD8+ T cells and mast cell inhibition, decreased angiogenesis, and reduced desmoplasia in multiple mouse models, resulting in reduced tumor size and increased survival rate (10, 23). In the phase III RESOLVE study, the combination of ibrutinib plus nab-paclitaxel and gemcitabine did not improve survival in patients without any previous cytotoxic chemotherapy for primary PC compared to chemotherapy alone (mOS, 10.8 months vs. 9.7 months, *P* = 0.323) (24). The phase II ACCEPT study demonstrated an mOS of 7.3 months in the afatinib combined with gemcitabine group compared to 7.4 months in the gemcitabine group (*P* = 0.80) (25). This was likely because the addition of targeted therapy to chemotherapy may have mitigated the ability to deliver the complete chemotherapy regimen and the tumor received a lower cumulative dose of all agents compared to patients in the chemotherapy alone group. In the phase II ACCEPT study, AEs were more frequent in the combination therapy group, which was consistent with the present study results showing a significantly higher toxicity burden in the combination group. In our study, we found that chemotherapy combined with targeted therapy was more prone to result in grade 3/4 leukopenia compared to chemotherapy alone. Family history of cancer was an independent mOS predictor for PC patients who received the chemotherapy alone treatment. In addition, the present research also showed that there was no statistical difference in mOS between single- and multi-agent chemotherapy (5.1 months vs. 7.9 months, *P* = 0.967) groups, which was consistent with the randomized phase III NAPOLI-1 trial results (26). The response in the NAPOLI-1 trial was less prominent in patients treated with nanoliposomal irinotecan with fluorouracil/leucovorin compared to fluorouracil/leucovorin as the third-line treatment (mOS, 5.4 months vs. 4.3 months, *P* = 0.178). Therefore, the appropriate use of low-intensity regimen in third-line antitumor therapy can also prolong survival. To our knowledge, no clinical studies have been carried out on third-line antitumor therapy to support this conclusion, which still needs to be confirmed by studies with a larger sample size.

The optimal therapy sequencing remains unknown and is largely defined by physician preference in practice. In our results, the order of gemcitabine- and fluorouracil-based regimens as the first- and second-line treatment pairs was not an independent predictor of third-line treatment OS, which is consistent with what has been reported so far. In the study by Jung et al., first-line palliative chemotherapy regimens and the order of subsequent chemotherapy regimens were not associated with survival outcomes in third-line treatment patients (27). Advances in systemic chemotherapy over the past decade have been limited, and the mechanism of chemotherapy resistance is still unclear. More trials will be carried out to explore the mechanism of chemotherapy resistance and provide credible data to identify the prognostic factors for chemotherapy rechallenge. According to our results, the order of chemotherapy drug treatment in the process of PC management can be selected according to the patient’s physical condition in the first- and second-line treatments. Patients can also receive personalized treatment.

The current study had limitations, as the research was performed at a single institution using retrospective analysis, and the number of patients included in each group was not balanced. First, the retrospective nature of the analysis may result in a potential selection bias such as the increase of survival would be due to the ECOG, and lack of medical records including molecular pathological information and immunohistochemistry may affect independent prognostic factor results. Second, therapeutic drug subgroups could not be analyzed further due to insufficient sample size. Third, the impact of different treatments after disease progression was not estimated in the study, which may affect the OS analysis. Nevertheless, our results are encouraging and support continued use and study of chemotherapy rechallenge to treat patients who fail to respond to the second-line treatment, as well as further optimization of selection of patients who are most likely to benefit.

## 5 Conclusion

Treatment with chemotherapy-based therapy as the third-line treatment combined with targeted therapy or immunotherapy failed to improve survival benefits and demonstrated higher safety risks. In particular, blood test results should be monitored more closely in patients undergoing multi-agent chemotherapy combined with targeted therapy or immunotherapy to prevent the occurrence of grade 3/4 leukopenia. The treatment order of gemcitabine- and fluorouracil-based regimens in the first- and second-line therapy does not affect third-line OS. Thus, in the first- and second-line therapy, the treatment order of chemotherapy drugs can be selected according to the patient’s physical condition. Therefore, patients who tolerate it should be treated mainly with chemotherapy. For patients who cannot tolerate chemotherapy, the use of targeted therapy or immunotherapy represents a survival benefit over supportive therapy. The targeted and immunotherapy drugs included in the present study were mixed, and more clinical trials are needed to explore the feasibility of chemotherapy-free treatment in advanced PC.

## Data availability statement

The original contributions presented in the study are included in the article/**Supplementary Material**. Further inquiries can be directed to the corresponding authors.

## Ethics statement

The studies involving humans were approved by the Ethics Committee of Zhejiang Provincial People’s Hospital. The studies were conducted in accordance with the local legislation and institutional requirements. The participants provided their written informed consent to participate in this study. Written informed consent was obtained from the individual(s) for the publication of any potentially identifiable images or data included in this article.

## Author contributions

H-RL and P-FZ contributed equally to this work. Z-LC, and LY revised the manuscript. All authors wrote and revised the manuscript and issued the final approval for the version to be submitted.
